# Excitation and Adaptation in Bacteria–a Model Signal Transduction System that Controls Taxis and Spatial Pattern Formation

**DOI:** 10.3390/ijms14059205

**Published:** 2013-04-26

**Authors:** Hans G. Othmer, Xiangrong Xin, Chuan Xue

**Affiliations:** 1 School of Mathematics, University of Minnesota, Minneapolis, MN 55455, USA; E-Mail: xinx0016@umn.edu; 2 Department of Mathematics, Ohio State University, Columbus, OH 43210, USA; E-Mail: cxue@math.osu.edu

**Keywords:** *E. coli*, Tar receptor, signal transduction, methylation, phosphorylation

## Abstract

The machinery for transduction of chemotactic stimuli in the bacterium *E. coli* is one of the most completely characterized signal transduction systems, and because of its relative simplicity, quantitative analysis of this system is possible. Here we discuss models which reproduce many of the important behaviors of the system. The important characteristics of the signal transduction system are excitation and adaptation, and the latter implies that the transduction system can function as a “derivative sensor” with respect to the ligand concentration in that the DC component of a signal is ultimately ignored if it is not too large. This temporal sensing mechanism provides the bacterium with a memory of its passage through spatially- or temporally-varying signal fields, and adaptation is essential for successful chemotaxis. We also discuss some of the spatial patterns observed in populations and indicate how cell-level behavior can be embedded in population-level descriptions.

## 1. Introduction

Most organisms have developed signal detection systems that extract information from their environment to enable them to find food and mates, initiate developmental changes, avoid harmful environments or execute any of the multitude of actions and behaviors in their repertoire. Since most organisms maintain a clear distinction between inside and outside, many primary environmental signals do not penetrate the organism very far, and therefore mechanisms for transducing an external signal into an internal signal, and where appropriate, an internal response are needed. For example, at the cellular level extracellular hydrophilic *first messenger* signals elicit a response via receptors in the cell membrane that transduce the signal into an intracellular *second messenger* signal. Similarly, in the sensory systems of higher organisms light or mechanical stimuli are transduced into an electrical signal that is processed at a higher level. The overall process from signal to response in *E. coli*, the model system described in detail later, can be summarized as follows.

**Figure:**
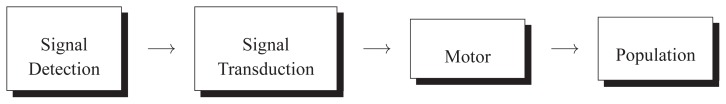


The response at the individual level to changes in the signal involves changes in the bias of the flagellar motor, and this can also lead to a response in the form of spatial pattern formation at the population level.

**Figure 1:**
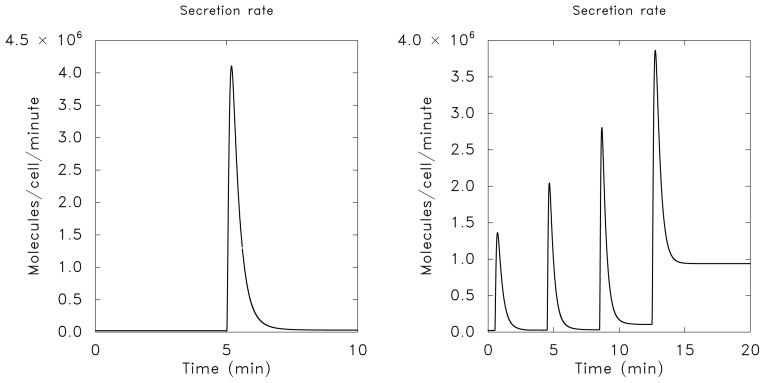
Two examples of the response of an adapting system to changes in the stimulus level. We show the predicted cyclic AMP (cAMP) relay response, as measured by the secreted cAMP, to extracellular cAMP stimuli in the cellular slime mold *Dictyostelium discoideum*. Left: A step change in extracellular cAMP from 0 to 10^−^^8^ M elicits a single pulse of secreted cAMP. Right: The system responds and adapts to a sequence of step increases ranging from 10^−^^9^ M to 10^−^^6^ M, but at the highest stimulus the transduction system saturates. (From [1], with permission.)

Signal transduction systems often filter the signal as well, since not all features of a signal are equally important. Often the important information in a signal is the short-term change in amplitude, rather than the absolute amplitude itself, and many systems have evolved to ignore constant background signals, yet remain responsive to changes in the signal. In such systems a step change in the signal from one constant level to another may elicit a transient change in one or more components of the internal state and some behavior of the organism, followed by a return to a basal level of that component or behavior. The process that leads to termination of the response in the face of a constant stimulus is called desensitization, habituation, or adaptation, depending on the context, but here we use adaptation when the stimulus does not provoke any gross rearrangements or alterations in the signal-processing machinery, whereas desensitization may involve structural changes such as the degradation of receptors. The visual system and mechanoreceptors in the dermis of mammals provide examples of adaptation to certain stimuli, but this capability is very common in sensory systems. In general adaptation also involves maintenance of sensitivity to further changes in the signal, and here we define an adapting sensory system as one that responds transiently to a transient change in the signal, returns to a basal activity level in the presence of a prolonged constant stimulus, and retains sensitivity to further changes in the stimulus. These characteristics are shown schematically for another cellular model system in Figure 1; detailed models of adaptation in *E. coli* will be discussed later. Clearly adaptation represents a form of memory, since having it in a signal transduction system enables the organism to avoid responding to a constant signal when such a response is not advantageous. In addition, by adapting to background levels of a signal (or equivalently, changing the sensitivity to the amplitude of signals) the sensory system can process a far greater range of amplitudes. In fact the range of signal amplitudes that can be tolerated is enormous. For example, the visual system in certain amphibians can detect and respond to light stimuli whose amplitude ranges over five or more orders of magnitude [2].

## 2. Signal Transduction in *E. coli*

At the cellular level and higher, the response to environmental signals frequently involves *taxis*, which is directed movement toward or away from an external stimulus. If it is toward the stimulus the taxis is positive, and otherwise it is negative. Many different types of taxis are known, including aerotaxis, chemotaxis, geotaxis, haptotaxis, and others. The purposes of taxis range from movement toward food and avoidance of noxious substances to large scale aggregation for the purpose of survival. The process by which a cell alters its speed or frequency of turning in response to an extracellular chemical signal is also frequently called chemotaxis, although it is more accurate to describe it as chemokinesis. Chemotaxis in this broader sense has been most thoroughly studied in the peritrichous bacteria *Escherichia coli* and *Salmonella Typhimurium*, particularly in *E. coli*. In this section, we first discuss the chemotactic behavior of *E. coli*, then describe the biochemical aspects of the signal transduction system. Later we discuss mathematical models and analysis of the system.

### 2.1. Cell Movement and Taxis

*E. coli* cells move by rotating rigid flagella in a corkscrew-like manner [3]. Each cell contains 6–8 flagella distributed uniformly over the cell surface, and when rotated counterclockwise (CCW), the flagella coalesce into a propulsive bundle, resulting in a relatively straight “run” [4]. When rotated clockwise (CW) they fly apart, resulting in a “tumble” which reorients the cell but causes no significant change of location. The cell thus alternates between runs and tumbles. In the absence of stimuli, the probability of a tumble is essentially independent of when the last tumble occurred [5]. The mean run interval is about 1 s in the absence of chemotaxis; the mean tumble interval is about 0.1 s [6]. Both are distributed exponentially, with shorter intervals more probable. The mean run length is 35 *μ*m [6], and the speed may range from 20 to 60 *μ*m s^−^^1^ [5]. Because of rotational Brownian movement, runs are not perfectly straight, and cells can veer off course by as much as 90° in 10 s [6]. The angles between two successive runs appear to be gamma distributed [7], with a mean of 68° in a medium of low viscosity and 103° in one of medium viscosity [7,8]. In the absence of chemotaxis, the diffusion constant of cells in liquid culture is around 4.8 − 5.2 × 10^−^^6^*cm*^2^*s*^−^^1^ [6,9].

A chemoeffector alters the probabilities that the flagella will rotate in a given direction, thereby changing the frequencies of runs and tumbles, and these probabilities change in response to *temporal* changes in the chemoeffector concentrations detected by the cell. A transient increase in the concentration of an attractant or a decrease in that of a repellent leads, after a 0.2 s. latency period, to an increase in the probability of counterclockwise rotation (*p*(*CCW*)) above the baseline probability of 0.64 [4]. For modest steps *p*(*CCW*) reaches a maximum at 0.4 s, crosses below the baseline at 1 s, reaches a minimum at 1.5 s, and returns to the baseline at about 4 s. A ramp or spatial gradient must exceed a threshold level in order to elicit a response [10]. A decrease in attractants or increase in repellents causes a decrease in *p*(*CCW*) [4], and the response is more rapid than that for a positive gradient. However, the response threshold for a negative gradient is large, so that *p*(*CCW*) remains at baseline for most negative gradients encountered in the wild [6]. When a gradient exceeds threshold, it is found experimentally that *p*(*CCW*) is proportional to the time derivative of the level of chemoreceptor occupancy, and this relationship holds for concentrations in a range near the receptor dissociation constant [10].

*E. coli* respond chemotactically to a variety of attractants and repellents over a range of concentrations which exceed a threshold concentration but do not saturate a cell’s receptors [6]. The response to aspartate may range over 5 orders of magnitude [11], with a threshold of 3 × 10^−^^8^ M [12] or 6 × 10^−^^8^ M [13] (depending on what medium and form of aspartate are used) and a peak chemotactic response at 10^−^^2^ M[13]. The response is sensitive to changes in aspartate occupancy of 0.1–0.2%, which corresponds to the binding of one or two ligand molecules per cell [11]. If we define the gain in signal transduction as the change in rotational bias divided by the change in receptor occupancy, the gain can be as high as 55 [14]. If we define the upstream signaling gain as the ratio of the relative change in kinase activity divided by the change in receptor occupancy, it is up to 35 [15].

### 2.2. The Biochemistry of Signal Processing in *E. coli*

**Figure 2:**
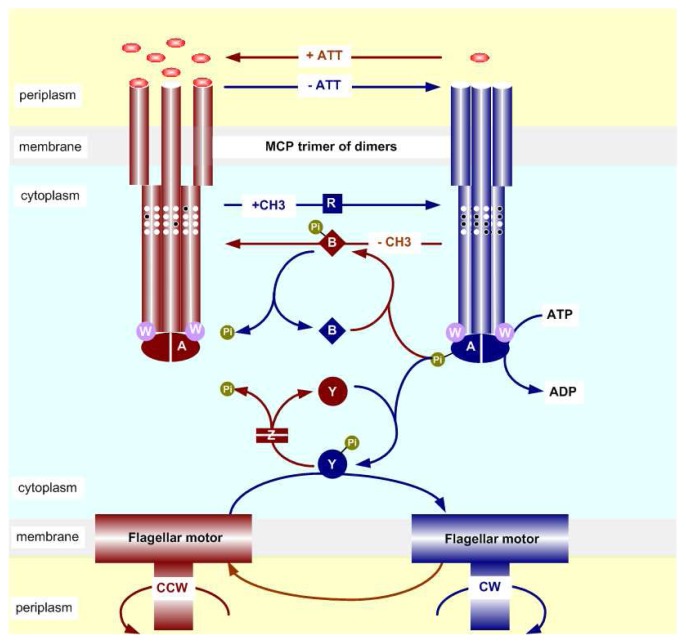
A schematic of the signal transduction pathway in *E. coli*. The trimer of chemoreceptor homodimers spans the cytoplasmic membrane, with a ligand-binding domain on the periplasmic side and a signaling domain on the cytoplasmic side. The cytoplasmic signaling proteins, denoted Che in the text, are identified by single letters, e.g., A = CheA. Proteins and reactions in red promote counterclockwise (CCW) rotation of flagella, and those in blue promote clockwise (CW) rotation of flagella. Receptor methylation sites involved in adaptation are shown as white (demethylated) and black (methylated) circles.

Two essential properties of the *E. coli* chemotaxis are excitation and adaptation, which stem from a signal processing system comprised of five chemoreceptor types–(Tsr - taxis to serine and repellents, Tar-taxis to aspartate and repellents, Tap - taxis to dipeptides, Trg-taxis to ribose and galactose, and Aer-taxis to oxygen) and six Che-proteins (CheA, CheW, CheY, CheZ, CheR, and CheB). The signal transduction pathway based on these proteins is depicted in Figure 2 and discussed below.

**Figure 3:**
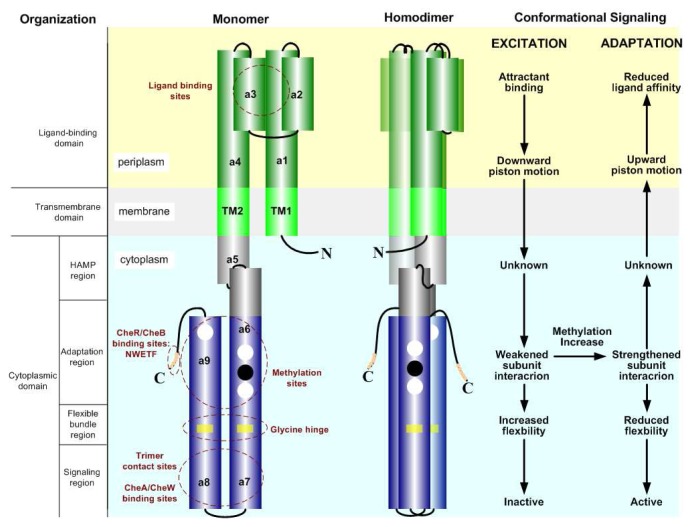
The structure of chemoreceptors. The schematic view of a chemoreceptor monomer (left) demonstrates the primary architecture consisting of ligand-binding domain (*α*1–*α*4), transmembrane domain (TM1–TM2), and cytoplasmic domain (*α*5–*α*9). The cytoplasmic domain can be further divided into four functional subdomains: the HAMP region, the adaptation region, the flexible bundle region, and the signaling region. The schematic view of a chemoreceptor homodimer (middle) illustrates the spatial organization, and the conformational changes of the homodimer involved in the excitation and adaptation phases are shown in the flowchart (right), summarized from [30].

Chemoreceptors are the transmembrane methyl-accepting chemotaxis proteins (MCP) that bacteria use to detect chemicals, light, or temperature. Among the five classes, Tsr and Tar are the major-type receptors with a few thousand copies per cell; Tap, Trg, and Aer are the minor types with a few hundred copies per cell. The functional form of chemoreceptors is a helical, intertwined homodimer. Each monomer consists of a variable periplasmic ligand-binding domain, a transmembrane domain, and a conserved cytoplasmic signaling domain. The ligand-binding domain contains a four-*α*-helix bundle (*α*1–*α*4 in Figure 3) arranged in parallel to form a cluster of eight helices in the dimer [16]. The helices *α*1 and *α*4 of the bundle extend to the helices TM1 and TM2 of the transmembrane domain respectively, and TM2 is linked to the cytoplasmic domain. The molecular symmetry generates two ligand-binding sites, each at the dimer interface within the quasi-four-helix bundle near the top of the molecule, distal from the membrane. Aspartate binding is negatively cooperative in that binding of an aspartate to either site causes an asymmetric change in the dimer that precludes binding at the second site.

The cytoplasmic domain extends from the transmembrane domain and bends back via a “U” turn (*α*5–*α*9 in Figure 3) [17]. This domain is highly conserved and the degree of sequence identity is maximal in the “U” turn region and declines away from the center [18,19]. The cytoplasmic domain consists of four primary functional regions: (1) histidine kinase, adenylyl cyclase, methyl-binding proteins and phosphatase (HAMP) region (*α*5 in Figure 3); (2) adaptation region, including two helixes (*α*6 and *α*9 in Figure 3); (3) flexible bundle region; and (4) signaling region (*α*7 and *α*8 in Figure 3). The structure of the HAMP subdomain is proposed as two amphiphilic helices joined by a connector in the monomer and a parallel, four-helix bundle in the dimer, which fits the role in converting ligand-binding conformational changes into intracellular signaling [20,21]. The subdomains (2)–(4) in the homodimer is a continuous four-*α*-helix, antiparallel coiled-coil containing two helixes from each monomer with a hairpin turn at its membrane distal end [17]. The adaptation region of each monomer contains four or more glutamyl residues, glutamate (E) or glutamine (Q), located midway along the coiled-coil (circles shown in Figure 3), which can be modified by the methyltransferase CheR and the methylesterase CheB [22–24]. These residues are spaced in heptad repeats along one face of each helix [25]. The flexible bundle region contains a conserved glycine hinge consisting of six glycine residues in a plane helix bundle in each monomer, which allows its long axis to bend 10° [26,27]. The region is known to be crucial for kinase control in that substitution of larger residues for glycine locks the receptor in the kinase-on or -off state [27]. The signaling region, bracketing the hairpin turn, is highly conserved and serves as a substrate for interaction with CheA and CheW [22]. The carboxyl terminus of Tar and Tsr carries a conserved pentapeptide (NWETF or NWESF) that binds with CheR and CheB [28,29].

In addition to the chemoreceptors, the excitation phase involves a two-component signal transduction system to controlmotor behavior, based on CheA, a histidine protein kinase (HPK), and CheY, a response regulator. HPK is linked to a sensory unit that detects changes in the environmental condition and when activated by the unit, the kinase catalyzes phosphotransfer from ATP to its own histidine residue. The response regulator, when phosphorylated by HPK, acts directly to modify the bias of the motor, and thereby leads to a change in cellular behavior. In *E. coli* CheA, which functions as a dimer, associates with receptors as well as with a monomeric protein CheW, which serves as a scaffold for receptor and CheA, to form stable ternary signaling complexes. The complexes sense environmental changes and regulate autophosphorylation of CheA in the presence of ATP. Attractant binding or repellent release inhibits the kinase activity; attractant release or repellent binding promotes it. CheY, reversibly bound to CheA, is phosphorylated by CheAp and then diffuses to the flagellar motors. CheYp binds to the protein FliM at the base of the motors and changes the rotational bias of the flagella, enhancing the probability of clockwise rotation (*p*(*CW*)) and therefore promoting the tumbling of the cell. The dimeric protein phosphatase CheZ assists in dissipating CW signals by forming CheYp–CheZ oligomer and enhancing dephosphorylation of CheYp. In *E. coli* and *S. typhimurium*, the gene *cheA* encodes two forms of CheA: the full-length CheAl, which plays an essential role in chemotaxis, and the short CheAs, which lacks the phosphorylation site [31]. CheZ binds to the N-terminus of CheAs and forms mixed oligomers, and the CheAs-CheZ complex formed *in vitro* shows a greater dephosphorylation activity on CheYp than free CheZ [31,32]. Therefore, CheAs contributes to recruiting CheZ to the signaling complexes and then CheZ-dependent localization of CheY [33,34].

The adaptation phase involves CheR and CheB, proteins involved in changes of the methylation level of chemoreceptors. CheR methylates glutamate (*E* → *E_M_*); CheB demethylates glutamate (*E_M_* → *E*) and deamidates glutamine to glutamate (*Q* → *E*). The activity of CheR is unregulated, whereas that of CheB is strongly enhanced upon phosphorylation by CheAp thus CheB is activated by feedback signals from the signaling complexes, which generates a negative feedback loop. The methylation level of a receptor affects the autophosphorylation rate of CheA, in that each addition of a methyl group increases CheA activity and each removal of a methyl group decreases CheA activity. Since methylation by CheR counteracts the effect of attractant binding or repellent release and demethylation by CheB counteracts the effect of attractant release or repellent binding, they are responsible for the relatively slow phase of adaptation to stimuli after the initial excitation phase. CheR is targeted to receptors through binding to the C-terminal pentapeptide sequence NWETF or NWESF that two major chemoreceptors Tar and Tsr contain, which the minor types do not [35]. The low-abundance receptors lack the docking site for CheR and are defective in methylation. They stimulate kinase only weakly *in vitro* and cannot support chemotaxis when expressed alone, but they mediate strong responses to stimuli in wild-type cells. One explanation could be that methylation occurs via an inter-dimer process. It has been found that CheR bound to one monomer in a Tsr dimer catalyzes the addition of methyl groups to a monomer in an adjacent dimer [36]. CheB also binds to the pentapeptide sequence, but with a much lower affinity than CheR. Interaction of CheB with the sequence activates demethylation by allosterically activating the receptor substrate and thereby increasing the reaction rate, whereas CheR binding at the sequence increases the enzymatic activity near the methyl-accepting glutamates [37].

The high signaling sensitivity and wide response range in *E. coli* chemotaxis probably stem from chemoreceptor clustering [38,39]. Though the exact organization of chemoreceptor clusters has not been determined unequivocally, it is known that chemoreceptors form stable homodimers [16,40–44], that three homodimers assemble into a trimer of dimers [17,45–48], that a large number of trimers cluster into an approximately hexagonal array [49–55], and that several arrays localize at the cell poles [38]. First, the trimer of dimers is formed through direct dimer-dimer interaction at the helical hairpin tips, and the trimer contact residues are identical in all five types of chemoreceptors [17]. The homodimers within a trimer can be either pure-type or mixed-type, which reflects the relative cellular abundance [46]. Receptors still form trimers of dimers in the absence of other chemotactic proteins [46], but without CheA or CheW, trimers exchange their dimer members, and in the presence of both, the exchanges do not take place [47]. Thus, CheA and CheW stabilize trimer assemblies, probably through binding interaction with receptors. However, overexpression of CheWinterferes with trimer formation, probably because bound CheW masks the trimer contact surfaces [47,56], and CheW competes for binding sites on receptors with CheA [57,58]. The stoichiometry of receptors, CheA and CheW in the ternary signaling complex has not been firmly established. Though several earlier studies reported different results [58–61], the recent studies presented the consistent stoichiometry of MCP : CheA : CheW = 6 : 1 : 1, suggesting that the ternary signaling complex is composed of 2 trimer of MCP dimers, 1 CheA dimer, and 2 CheW monomers [54,55,62]. An *in vivo* study estimates the stoichiometry as 3.4 receptor dimers/1.6 CheW monomers/1 CheA dimer, suggesting that the ternary signaling complex is composed of one trimer of MCP dimers, one CheA dimer, and two CheW monomers [61]. Secondly, in respect to the larger patches of MCP-CheA-Chew signaling complexes that are roughly hexagonally packed, about 80 percent are located at one or both cell poles and the rest are distributed in non-polar, lateral patches at future division sites [63]. The polar patches are mobile within the curved membrane of the pole, and the lateral patches are fixed [63]. The patches appear circular or ellipsoid with varying sizes and an average diameter 250 nm [61]. The patch size is not variable with the expression level of MCP, CheA and CheW, and the packing density is slightly variable with the culture conditions [53]. Recently, two imaging studies show that the hydrophobic interaction between CheW and CheA (the P5 regulatory domain) connects the trimer of dimers into an extended hexagonal receptor array [54,55]. Lastly, as to polar localization, knock-out of CheA or CheWreduces the number and size of polar patches, especially CheW, while CheY, CheZ, CheB and CheR are not required [38,64]. Localization and clustering seem independent since the minor receptors Trg and Tap are deficient in clustering if locked in the state of fully inhibited CheA, but polar localization is not altered [65].

With the preceding description of the structure of the chemoreceptor clusters, which involve multiple levels of organization, at hand, we discuss the structure-function relationship (summarized in Table 1). The receptor homodimer is the minimal stable structural unit of receptor clusters. A single homodimer is able to perform ligand binding, signal transmission from periplasm to cytoplasm, and adaptational modification, all of which are not dependent on the dimer-dimer interaction, but fails to perform CheA kinase activity control and thus chemotactic response [48,66]. The trimer of dimers is a core structure in chemoreceptor clusters and plays a central role in the signaling function. It is structurally the smallest stable signaling complex when bound with CheA and CheW [17,45–47], and serves as the architectural unit of the larger receptor array [49–53]. Functionally, the trimer of dimers is the minimal signaling unit, and is necessary for most of the functions (Table 1). The homodimer fails to control CheA kinase activity, and compared to other larger clusters, the trimer of dimers has the maximal kinase activation [48,66]. The interaction among dimers within a trimer is probably more important for the signaling function than the longer-range interaction among trimers, considering that the extremely high cooperativity of receptors (the Hill coefficient measured in kinase activity responses is larger than 3) is only observed in two special cases, the responses by the *cheRcheB* mutant cells with Tar or Tsr highly overexpressed [39] or by the receptor Tsr *in vitro* [67], while in wild-type cells and other *cheRcheB* mutant strains, the cooperativity is moderate (the Hill coefficient is less than 3). A recent study on CheW provides indirect support for the central role of the trimer of dimers, in that chemotaxis is reduced in cells with CheW overexpressed, because the excess level of CheW prevents trimer formation [56]. In evolutionary terms, the structural unit of a trimer of dimers and the underlying signaling mechanism are highly conserved and could be a universal architecture for many bacterial species [52].

Finally, we discuss the conformational aspect of the signaling mechanism of receptor clusters. A homodimer is usually treated as a two-state (active or inactive) switch. Ligand binding, methylation/demethylation, and interaction with neighboring receptors can shift the equilibrium-between the two signaling states in a dimer. In more detail, attractant binding initiates a piston-like sliding of the transmembrane signaling helix (TM2 in Figure 3) towards the cytoplasm along its long axis perpendicular to the membrane, by ~ 0.15 nm [68], and adaptional modification reverses the motion and drives the movement towards the periplasm. The conformational signaling of the HAMP region is not clear yet, but presumably it interconverts the ligand binding-induced sliding of the transmembrane helix and the conformational change of the adjacent adaptation region. The signal conversion should not involve large helical displacements in that the HAMP domain, which is constrained by disulfides across the two helices or the adjoining subunit interface, still transmits the attractant-generated signals [21,44]. The conformational signaling of the adaptation region involves weakening or strengthening the subunit interactions of the domain corresponding to the off or on state, respectively. The ligand binding-induced signal changes the subunit interface (destabilized by attractants and stabilized by repellents) through mechanical forces [44,69], and methylation/demethylation alters the interface via electrostatic forces [70]. Specifically, the glutamates and several other side chains located in the interface are anionic, and covalent neutralization of them by methyl esterification or amidation stabilizes the interface and activates the kinase. The flexible bundle region enables the cytoplasmic domain to bend and/or twist, increasing (kinase deactivation) or decreasing (kinase activation) its flexibility. The conformational change of the signaling region is still under investigation, but like the adaptation region, the subunit interface seemingly plays an important role here in that some mutations at interfacial locations lock the receptor in the active state [44,71]. When extending conformational signaling to the scale of trimers of dimers, a recent study proposes a two-state model for kinase control (trimer expansion or closing, corresponding to kinase deactivation or activation, respectively) [68]. Attractant binding drives piston sliding of the transmembrane helix in one dimer member of a trimer, then the displacement induces bending of the dimer around the HAMP domain, and finally the trimer of dimers expands, leading to kinase deactivation. Two other conformational changes have been suggested to take place when the trimer of dimers changes states [30], rotation of the periplasmic domain of each dimer about its long axis [72], and tilting of dimers relative to the central trimer axis [73,74], both of which could be driven by the change in flexibility of the four-helix bundle in the cytoplasmic domain [27,70]. With respect to the larger receptor clusters, the mechanism remains open. The advanced imaging for chemoreceptor arrays by cryoelectron tomography suggests that the interconnected CheA and CheW proteins might serve the molecular basis for the conformational spread throughout the receptor array of an attractant signal originating at one MCP dimer [54,55]. A more comprehensive review is given in [30]; we turn next to some general considerations about excitation and adaptation and then describes mathematical models of signal transduction in *E. coli*.

## 3. Models of Signal Transduction and Adaptation

The chemotaxis signal transduction system in *E. coli* must cope with a wide range of changes in ligand levels that are transduced into the bias of the flagella via the level of CheYp. It was predicted theoretically [75] and later verified experimentally [76] that much of the observed gain arises either in the interaction of CheYp with the motor, or from interaction between motor subunits. As a result, small changes in CheYp are strongly amplified in either or both of these steps, and therefore it is necessary that CheYp return to a level close to its prestimulus value after a change in ligand concentration. Otherwise there would only be a narrow range of ligand concentrations over which the bias does not saturate (at one or zero). Thus to maintain both sensitivity and responsiveness to a wide range of ligand levels, it is necessary that the tumble signal adapt, *i.e*., to return to a level very close to its prestimulus value, and if it returns to exactly its prestimulus level for all ligand concentrations we say that it adapts perfectly.

**Figure 4:**
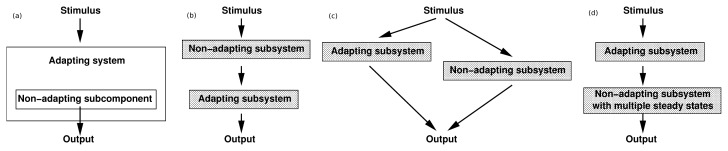
Examples of various adapting and non-adapting systems. (**a**) A signal transduction pathway in which a specified upstream quantity adapts, but the output species further downstream does not, because the output depends on a non-adapting subcomponent of the upstream adapting quantity; (**b**) Similar to that in (**a**), except that here the upstream quantity does not adapt, but the subcomponent adapts, and so the output species adapts as well. We will see later that one may think of the adapting subcomponent as the sum of the states of the receptor containing phosphorylated CheA, and the upstream non-adapting quantity as some other function involving the various phosphorylated and unphosphorylated states; (**c**) An example of a signal transduction pathway in which a specified upstream quantity adapts, but the output species further downstream does not, because the output depends on both the adapting quantity and another non-adapting quantity; (**d**) An example of a signal transduction pathway in which a specified upstream quantity adapts, but the output specified further downstream does not because it depends on an intermediate subsystem which possesses more than one stable steady state. Transient changes in the upstream quantity may cause the intermediate subsystem to reach a steady state different from its prestimulus state.

This raises the question as to how adaptation can be ensured in a system of chemical reactions. To illustrate that this is not easy to answer in general, we observe that for any network of chemical reactions, adaptation of a given component does not necessarily ensure adaptation of a particular species located further “downstream” in the kinetic pathway. This is demonstrated with several schematic counterexamples in Figure 4.

In addition to the problem inherent in specifying an upstream adapting quantity *a priori*, some models invoke unwarranted restrictions on the system’s kinetics in order to facilitate calculation of the steady state levels of the upstream quantity. Goldbeter and Koshland [77] assume that a receptor cannot be both methylated and free of ligand at the same time. Asakura and Honda [78] likewise assume that certain receptor states cannot be attained, and further assume that the ratio of the methylation and demethylation rates is the same for each methylation state, that a receptor generating a tumble signal cannot be methylated, and that an attractant-bound receptor can neither be demethylated nor generate a tumble signal, regardless of its methylation state.

### 3.1. Adaptation in Model Systems

In view of the fact that *ad hoc* assumptions may lead to models with limited applicability, we present a method for determining relations among rate constants which, where applicable, ensures that perfect adaptation occurs. The method is applicable to a variety of chemical systems, requires no *a priori* assumptions regarding a second adapting quantity, and places few restrictions on the kinetics. By perfect adaptation, we mean that there is a species in the transduction pathway whose concentration changes transiently in response to a change in the level of some stimulus, but whose steady state concentration is independent of the stimulus level. Our analysis deals with the question of what guarantees that the quantity in question returns to its basal level; the question of whether the transient response is suitable must be answered a case-by-case.

As we will see later, the signal transduction system in a single bacteriuum can be described by a finite number of state variables and an evolution equation that determines how the state changes under prescribed inputs or stimuli. We denote the state vector by *u*(·) ε *R^n^* and write the evolution equation in the form

(1) $$\frac{du}{d\tau }=F(u,S)$$

where *S* ε *R* represents the stimulus or input to the system. In general a change in *S* leads to a change in the transient and steady-state values of *u*, but in systems that adapt, some functional of *u* should be independent of *S* when *S* is time-independent. Thus suppose that the response ℛ of the system is a functional 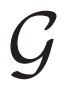 of the state *u* given as follows:

(2) R(τ)=G(u(τ))

More generally, 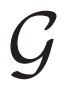 could depend on the derivatives of the state variables, their past history, or directly on the stimulus and its derivatives. If we only consider systems whose “basal dynamics” are time independent, which means that the system has an asymptotically stable steady state in the presence of any constant stimulus, we can define perfect adaptation to constant stimuli as follows.

**Definition 1***The response ℛ of a system whose dynamics are governed by Equation (1) is said to adapt to constant stimuli if the steady state response is independent of the magnitude of the stimulus S.*

Evidently this definition allows for the trivial case when *F* is independent of *S*, in which case there is no change in response to any changes in *S*. Furthermore this definition of adaptation does not imply that the steady state values of all variables must be independent of *S*, and in fact some of the state variables must generally change to compensate for the stimulus changes. In the case of *E. coli* the methylation level compensates for the background signal level, and thus does not adapt. The reader can consult [75,77,79–81] for a review of models that involve adaptation, including some for bacterial chemotaxis and adenylyl cyclase. A very general result that defines the structure necessary in a dynamical system in order that it can adapt is given in [82].

A widely-used model system that illustrates some of the essential features of an adaptive system is given as follows. Suppose that there are two internal state variables *u*_1_ and *u*_2_, and that these variables evolve according to the following equations.

(3) $$\begin{array}{l} \frac{du_{1}}{d\tau }=\frac{f(S(\tau ))-(u_{1}+u_{2})}{\tau_{e}}\\ \frac{du_{2}}{d\tau }=\frac{f(S(\tau ))-u_{2}}{\tau_{a}} \end{array}$$

In these equations the function *f*(·) encodes the signal transduction steps, and it should have the property that *f*(0) = 0. For concreteness we suppose that the response is proportional to *u*_1_, *i.e.*, 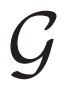 (*u*(*τ*)) = *au*_1_(*τ*) where *a* is a constant. Then this simple scheme can be viewed as having two input pathways, an excitatory one in which the stimulus increases the production of *u*_1_ and hence increases the response, and an inhibitory one that increases the production of *u*_2_, which in turn shuts off the response.

Since this system is linear, the solution can be obtained by quadrature once the stimulus is specified. For the special case in which *u*_1_(0) = *u*_2_(0) = 0 and *S*(*τ*) is a step function of amplitude *S*_0_ that turns on at *τ* = 0, the solution is as follows.

(4) $$\begin{array}{l} u_{1}=\frac{f(S_{0})\tau_{a}}{\tau_{a}+\tau_{e}}(e^{-\tau /\tau_{a}}-e^{-\tau /\tau_{e}})\\ u_{2}=f(S_{0})\;(1-e^{-\tau /\tau_{a}}) \end{array}$$

Thus the response occurs on two time scales, the scale of excitation, which is characterized by *τ_e_*, and the scale of adaptation, which is characterized by *τ_a_*. From this one sees that if *τ_e_<< τ_a_*, then whenever *τ >> τ_e_*, *u*_1_ relaxes to

$$u_{1}\sim f(S_{0})e^{-\tau /\tau_{a}}\equiv f(S_{0})-u_{2}(\tau )$$

**Figure 5:**
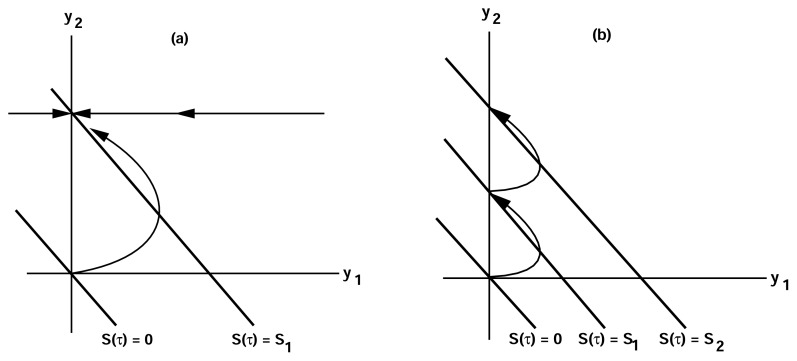
The response to single (**a**) and multiple (**b**) steps in the signal for the model adapting system described by Equation (3) when *f* is a linear function.

This is just the pseudo-steady-state value of *u*_1_ which is gotten by setting *du*_1_*/dτ* = 0. On the other hand, if *τ_a_<< τ_e_* then adaptation is rapid compared to excitation, *u*_1_ never rises significantly above zero, and there is no significant response. The typical response for a single step in the stimulus when *τ_e_< τ_a_* is shown in Figure 5(a), where one can see that when the system begins at (*u*_1_*, u*_2_) = (0*,* 0) neither *u*_1_ nor *u*_2_ exceed *S*_1_. The response to two step changes that are well separated compared to the adaptation time are shown in Figure 5(b).

We note from Equation (3) that when the stimulus *S*(*τ*) is constant the steady state level of *u*_1_ is zero, *i.e.*, the response adapts perfectly to any constant stimulus, but the level of *u*_2_ does not adapt. Moreover, when *τ_e_<< τ_a_* the system is excitable in the following sense. The rest state in the absence of a stimulus ((*u*_1_*, u*_2_) = (0*,* 0)) is asymptotically stable, but a brief stimulus of the proper type can produce a significant response, followed by a return to the steady state. Thus if *f* is linear, if the system is initially at (0*,* 0), and if *S*(*τ* ) = *S*_1_ for *τ* ε (0*, τ_e_*) and zero thereafter, then *u*_1_ will rise to approximately 2*S*_1_*/*3 and then return to zero. Usually an excitable system is considered as one that has a threshold and shows an *all-or-nothing* response, such as the firing of a neuron, depending on the magnitude of the stimulus. In contrast to this, the response of the present system is *graded* in that there is a response to any stimulus level. Other excitable systems that show a graded rather than an all-or-none response occur in models of intracellular calcium dynamics [83–85].

This simple model illustrates some of the basic features necessary in an adapting system, but there is no explicit biochemical basis for it. However the excitation variable *y*_1_ can represent the active state of a ligand-occupied receptor, whereas the adaptation variable could represent an internal variable that desensitizes the receptor. Of course the actual physical quantities should remain non-negative. A more realistic four-dimensional model, which is sometimes called the adapting box model, was first proposed and analyzed by Katz and Thesleff [86] in a study of adaptation produced by acetylcholine at the motor end-plate of frog muscle, and more general forms were subsequently used by others in a similar context ([87] and references therein). More realistic models for *E. coli* are described in the following sections.

## 4. Models of Signal Transduction in *E. coli*

*E. coli* chemotaxis has been the subject of various mathematical modeling studies since the early 1970s (Table 2). In recent work the focus of the models has changed from the basic excitation and adaptation properties of the signal transduction pathway to other properties of the system, such as receptor clustering-induced cooperativity and sensitivity. The modeling methods have ranged from classical chemical kinetics to statistical mechanics, and the simulation techniques used have varied from numerical methods for deterministic differential equations to Monte Carlo methods for stochastic processes. Quantitative modeling has played a significant role in understanding this system by providing a framework for interpreting existing data and stimulating new experiments, with the result that the multitude of experimental results are beginning to fit into a coherent picture.

We first focus on theoretical studies of the excitation and adaptation characteristics of the signal transduction pathway and the underlying mechanism for system robustness. The early modeling studies were directed toward understanding the observed adaptation in bacterial chemotaxis [4,10,88,89]. Macnab and Koshland [90] proposed a conceptual network for chemotactic responses in which a response regulator is upregulated by a fast enzyme activity and downregulated by a slow enzyme activity, and later Koshland proposed that methylation/demethylation of receptors was probably the source of the slow enzyme activity [89]. Block *et al.* [4] postulated a two-state assumption for the system and then formulated a deterministic model that includes a description of adaptation [10]. Goldbeter and Koshland proposed the first adaptation model that includes the ligand binding and one-site methylation reactions [91], and Segel and Goldbeter modified the model by allowing receptor modification to occur on both ligand-free and ligand-bound receptors [92]. Asakura and Honda [78] extended Goldbeter and Koshland’s model to multiple-site methylation. Bray *et al.* [93] first modeled the excitation response of chemotaxis using a simplified network without methylation and demethylation and later added a hypothetical reaction network for formation of the ternary MCP/CheA/CheWsignaling complexes [94]. Hauri and Ross [113] developed a model of the signal transduction pathway that exhibits both chemotactic excitation and adaptation to attractants and repellents. Spiro *et al.* [75] developed a model of the complete pathway based on three methylation states that included the phosphotransfer steps, and showed that the model accurately reproduces the step, ramp and saturation responses to aspartate on the correct time scales. That model was used predict that a high Hill coefficient ≤ 11 in binding of CheYp to FliM is needed to explain a modest gain 3 to 6 in the absence of cooperativity upstream in the signal transduction pathway, and this was later confirmed experimentally [76].

A long-standing question is how biological systems maintain the stability of their functions in the face of perturbations of parameters or state variables such as reaction rates or molecular concentrations. Barkai and Leibler [96] constructed a signal transduction model that includes the ligand binding and methylation/demethylation reactions among three components (MCP, CheR and CheB), and the model shows robustness of the ratio of adapted steady-state system activity over prestimulus activity. Following earlier two-state models for the receptor, they made the key assumptions that each receptor is either active or inactive, and that CheB acts only on active receptors at a rate that is independent of ligand binding. The direct coupling between kinase activity and demethylation rate provides an integral feedback control in their model and leads to robust perfect adaptation, as was demonstrated later [81]. However, the robustness of the output of the system such as the concentration of CheYp or the rotational bias of flagellar motors was not addressed since CheY was not included in the model. Later, an experimental study showed that the working range of the concentration of CheYp for the proper response of flagellar motors is so narrow that the level of CheYp in adapted cells can vary only about one-third from its optimal value [76], which indicates that the stationary concentration of CheYp should be tightly controlled. Kollmann *et al.* [111] showed that the signaling network topology of *E. coli* chemotaxis (with only a single methylation site considered) is robust to the intercellular variation in chemotactic protein concentrations arising from gene expression, and the variation of CheYp is much smaller than that of other proteins, and this has been confirmed by the experimental finding that the cells maintain the concentration of CheYp in the right range and still remain chemotactic upon up to 6.6-fold overexpression of all proteins in the system [111]. It was also reported that the fine-tuned adaptation systems [75,113] behave differently than the robust adaptation system [96], when examining the effects on the level of CheYp and the motor bias by the coordinate overexpression of all seven *che-* genes [114]. Other analyses, both deterministic and stochastic, are described in Table 2.

A comparison of the fine-tuned and the robust adaptation models shows that the significant difference lies in the treatment of methylation by CheR and demethylation by CheBp especially the latter. In the fine-tuned systems [75,113], CheR and CheBp can modify receptors in all states, while in the robust ones [96,99,111,116], CheBp can only access receptors in the active state and CheR in the inactive state (except in [96], where CheR is assumed to work at saturation in a constant rate on all receptors). Though a mechanism of the receptor activity-dependent methylation/demethylation leading to robustness of adaptation has been proposed theoretically [81,111], it remains to confirm it experimentally.

Next we review a large number of models that employ a receptor clustering-based explanation for the high signaling sensitivity, large transduction gain, and wide dynamic range of the signaling system. Bray *et al.* [118] proposed that receptor clustering could account for the observed sensitivity and dynamic range. Each receptor existing in an extended lattice interacts with its neighbors and conformational spread occurs from ligand-bound receptors to neighbors so that at low concentrations, deactivation of a single receptor is magnified and can inhibit the phosphorylation cascade to the flagella, while at high concentrations, the methylation/demethylation-induced adaptation can free those affected receptors to respond to further changes. Thereafter, many models have emerged, and we separate them into two categories.

### Category I

The models in this category are based on the hypothesis that receptors exist in an extended, weakly-coupled lattice network and use the Ising-type framework. As an implementation of Bray’s idea [118], Shi and Duke first adopted the Ising model developed for ferromagnetism to the chemotactic signaling. This application was based on the similarity of the two systems, in that each unit of the lattice lies in two stable states, active or inactive, that the probability of being in a state state is determined by not only its own properties but also the states of its neighbors, and that the ligand input could be treated as a magnetic field [97]. A Monte Carlo simulation of the model shows that a two-dimensional lattice of coupled receptors generates the higher sensitivity to external stimuli and the wider functional range of ambient ligand concentrations than an array of independent receptors [119]. Shi extended the previous model [97] by incorporating the effect of CheR and CheBp, and made a perfectly-adapting Ising model [98]. Then Shi compared the theoretical predictions of his models [97,98] with the experimental measures and found good agreement on the ratio of attractant binding to receptor-receptor interactions, the adaptation time, as well as the ratio of pre- and post-stimulus CheA phosphorylation [120]. Following that, Shi incorporated the effect of receptor movement into the model and showed that the receptor correlation remains strong for nearby receptors and decays exponentially with increasing distance between receptors [121]. Shimizu *et al*. incorporated the Ising-type description of receptor clustering into the stochastic model they developed earlier [99], compared the effects of size and geometry of receptor arrays, and convincingly showed the enhanced signal gain through receptor-receptor interaction [123]. Mello and Tu proposed a deterministic version of the Ising-type lattice model, taking into account receptor interactions among different species Tar and Tsr, and including methylation/demethylation [100]. Later, the mean-field theory was applied to simplify the model and also the Monte Carlo simulation was implemented [124]. Both models reproduced Sourjik and Berg’s FRET data on the *cheR/cheB/cheRcheB* mutant strains as well as the wild-type cells [15], although using two different parameter sets.

### Category II

These models hypothesize that receptors exist in several strongly coupled clusters and use the Monod-Wyman-Changeux (MWC)-type framework. The FRET experiments in Sourjik and Berg [39] indicate that the clustered chemoreceptors work in high cooperativity and the functional interaction mimics the behavior of multi-subunit allosteric proteins, and thus they proposed to use the classical MWC model [125] to explain it. Subsequently, Mello and Tu reported a generalized MWC model for allosteric interaction and multiple signal integration in heterogeneous receptor clusters, and it reproduces the measured responses for 14 mutant strains with varied expression levels of Tar and/or Tsr [107]. Further, the authors proposed a simplified version for homogeneous receptor complexes and studied the underlying mechanism of how the cells maintain high sensitivity over a wide range of backgrounds [126]. Recently, the Wingreen group pointed out that the differences of FRET data between wild-type, *cheR*, and *cheRcheB* mutant cells suggest two regimes of receptor behavior: regime I is characterized by low to moderate kinase activity and a low, constant inhibition number for half-maximal activity *K_i_*, in which coupling of receptors leads to high sensitivity (in the case of wild-type and *cheR* mutant cells); regime II is characterized by high kinase activity and a high *K_i_*, increasing with the methylated level of receptors, in which coupling leads to high cooperativity (in the case of *cheRcheB* mutant cells) [106]. Following the modified MWC framework and inspired by the recent experimental finding “assistance neighborhoods” that CheR and CheB can access five to seven receptors when tethered to a particular receptor [105], Endres and Wingreen extended the Barkai and Leibler model [96] to the mixed Tar-Tsr clusters and showed that “assistance neighborhoods” are necessary for precise adaptation because the probability of the enzymes CheR and CheB encountering fully methylated or demethylated receptors, which is believed to induce imprecise adaptation, is greatly reduced due to a large number of modification sites available [110]. Recently, Hansen *et al*. extended the ‘assistance neighborhood’ model by including binding and unbinding of CheR and CheB so that the authors could further consider the adaptation limits from the angle of CheR and CheB kinetics [127]. Using a similar model, Meir et al. analyzed the characteristics of precise adaptation and found the asymmetries (*i.e*., different adaptation time) in responses to addition and removal of attractants, and proposed two possible resources of the asymmetry: (1) dynamic phosphorylation of CheB and (2) scarcity of methylation site [128]. To dissect the Ising-type and the MWC-type models, the Wingreen group also compared the activity response of coupled receptors from three different models: a one-dimensional Ising-type model for a weakly-coupled receptor lattice, a two-dimensional Ising-type model for weakly-coupled receptor lattice, and a two-regime MWC-type model for isolated strongly-coupled receptor clusters. They found that Sourjik and Berg’s FRET data of activity responses to steps of chemoattractants for wild-type and *cheR* mutant strains [15] are inconsistent with the Ising-type model, but consistent with the MWC-type model, which seemingly suggests that receptors form isolated strongly-coupled clusters [129].

The models addressed above, either of Ising-type or MWC-type, treat the receptor homodimer as the basic functional unit and thus the receptor interaction is at the dimer-dimer level. However, it is now established that the trimer of homodimers serves as the core unit in signaling, especially in kinase control [48]. An increasing number of models concentrate on this unique structure. Albert *et al.* [102] developed a dynamic trimer formation model to account for high upstream sensitivity, which assumes that the time scale of association and dissociation of a trimer of dimers is comparable to that of ligand binding and kinase activity. The model produced good agreement with experimental data, but later the their assumption was disproved in that the half-life of trimers was experimentally estimated to be around 5 min [103] and thus a static scheme is more appropriate. Rao *et al.* reported a MWC-type model of the static trimer of dimers [130] and later Endres *et al*. reported another static trimer model [131] following the modified MWC framework [106]. The two models mainly explain *in vitro* kinase activity data on Tar [104,108,109] and Tsr receptors [67]. Park *et al*. performed sensitivity analysis for the trimer of dimers-based signaling [132].

Thus far the MWC-type models reviewed all have a prescribed stoichiometry and a fixed size of receptor clusters. In cells, the number of the receptor complexes involved in chemotactic responses probably varies with the stimulus magnitude. Recently, theoreticians have begun to develop models for dynamic signaling receptor clusters with a variable size. Endres *et al*. used a backward approach, determining the sizes of the signaling clusters through best fitting *in vivo* FRET data with the model [106], and found that the size increases with the methylation level, up to 2–3 fold [133]. Intrigued by this finding, Hansen *et al*. presented a model of dynamic signaling clusters of trimers of dimers, the boundaries of which are able to change during a simulation course, and the model shows that the active trimers of dimers seem to couple more strongly than inactive ones [134]. In a very recent study, a cutoff distance was used to determine the range of interacting receptors and the simulation on size-varied signaling clusters was performed to explore the effect of lateral density of receptor arrays on signaling sensitivity [53].

Next we discuss several models analyzing other features of the system. Lipkow *et al.* [135] did a stochastic simulation of the downstream pathway, including CheY phosphorylation, CheY/CheYp diffusion, CheYp binding to FliM and dephosphorylation, which has two important features (1) it incorporated diffusion of molecules in the stochastic simulation of chemical reactions and (2) it tracked the spatial locations of individual molecules in 3D. The model shows that when CheZ is restricted to receptor ends, the concentration of CheYp is constant throughout the cytoplasm, but when CheZ is free to diffuse, CheYp has an exponential gradient across the length of the cell, highest at the anterior end [135]. Later, Lipkow used the model to study the effect of CheZ localization and noted that clustering of the CheZ, CheYp and CheAs complexes at the cell poles introduces a negative feedback to maintain the CheYp level, which could play a secondary adaptation role and explain the overshoot of CheYp in *cheRcheB* mutant cells [136]. Recently, Endres [137] addressed the question of what determines the size of receptor clusters at the cell poles. The receptor cluster-membrane elastic energy disfavors large clusters due to their high intrinsic curvature, while the receptor-receptor coupling favors large clusters. The author hypothesized that the cluster size is determined by minimizing the cluster-membrane free energy and developed a free energy-based model for formation of clusters of trimer of dimers. Besides *E. coli*, Rao *et al*. modeled bacterial chemotaxis in a different species *Bacillus subtilis* and argued that the core control strategy of the two signaling pathways remains the same [138]. The modeling for the signaling pathway of *Rhodobacter sphaeroides* chemotaxis has begun and is becoming a new model system [139–141]. For further reading we recommend a comprehensive review on mathematical modeling of bacterial chemotaxis in 1976–2006 in Tindall *et al.* [144].

**Figure 6:**
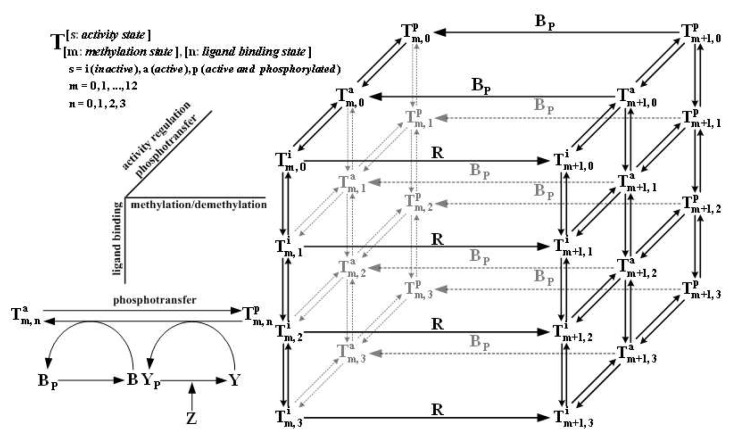
Signal transduction network. The basic unit of the network is the signaling complex, denoted by T. The three indices used to denote the properties of the complex are shown in the upper left corner. In the reaction network, vertical transitions are ligand binding and release, horizontal transitions are methylation and demethylation, and front-to-rear and reverse transitions are kinase activation, deactivation, phosphorylation and dephosphorylation. The details of the phosphotransfer transitions are depicted at the left. Adopted with permission from [143].

Lastly, we introduce a recent, trimer of dimers-based model [143]. The accumulating evidence that demonstrates the key role played by the trimer of dimers in signaling function for bacterial chemotaxis, especially Boldog *et al*.’s experiments showing that homodimers fail to regulate kinase activity and trimers of dimers perform the maximal kinase activation compared with other higher-order structures [48], suggested further study of the dynamics of the molecular structure. The existing models for the single trimer of dimers restrict to the equilibrium behavior and only consider the ligand binding and kinase activity control reactions, excluding the downstream phosphoryl transfer and methylation chain [130–132]. In contrast, we treat the ternary complex of one trimer of receptor dimers, one CheA dimer, and two CheW monomers as a signaling complex (depicted in Figure 2), incorporate the sensing unit into the overall signal transduction pathway (shown in Figure 2 and the corresponding transition network shown in Figure 6), and simulate the dynamics of the network in response to multiple stimuli [143]. The single trimer model could explain the kinase activity variation with ligand concentration for the methylation-fixed receptors Tar [104,108,109] and Tsr [67]*in vitro* and the CheYp responses in *cheRcheB/cheR/cheB* mutant cells *in vivo* [15]. We then did a sensitivity analysis for each stage of the signal transduction pathway and showed the enhancement of upstream sensitivity in ligand binding and activity regulation due to the structural assembly from homodimers to trimers of dimers. We also tested the robustness to model parameters and found that precise adaptation is well-preserved when varying the expression levels of protein components in individual or concert. However, in such cases the steady-state level of CheYp is not invariant and the adaptation time is also variable. Therefore, we make several new predictions as to how the adaptation time and the CheYp level vary with the quantity of signaling proteins. Because of the complex network and various states, the full model consists of 158 ODEs. To facilitate computation and further analysis, we do model reduction by applying multi-time-scale analysis and mean-field theory and simplify it into the 16-ODE and 4-ODE systems, respectively, while keeping the key features intact.

The single trimer model cannot reproduce the higher receptor cooperativity, *i.e*., the Hill coefficient of response larger than 3, and fails to explain the data of the *cheRcheB* mutant cells with Tar or Tsr highly overexpressed [39]. It suggests that the trimers of dimers are not independent of each other and that larger receptor clusters with trimer-trimer interaction must be involved. Here we extend the single trimer model to a trimer cluster model. We first consider the pure-type trimer of dimers (all three homodimers are of the same receptor type). For a given methylation level, a trimer of dimers has two activity states and four ligand-binding states. Therefore, a trimer of dimers at a fixed methylation level could exist in eight distinct free-energy levels. We denote the free-energy level for the active ligand-free trimer as $E_{on}^{m}$, and similarly the free-energy level for the inactive ligand-free trimer as *E_off_*. All energies are in unit of the thermal energy *k_B_T*. For simplicity, we specify $E_{on}^{m}$ as a function of the methylation level *m* and *E_off_* independent of *m*, since only an offset energy, the relative difference between the two energy levels, appears when use Boltzmann’s law to derive the formula for the probability of each state. Next, we deal with the ligand-binding states. We consider the first ligand binding to, or releasing from, an active trimer. In the steady state,

(5) $$\frac{3k_{1}^{on,m}L}{k_{-1}^{on,m}}=e^{-\Delta E_{1}^{on,m}}$$

$\Delta E_{1}^{on,m}$ is the free-energy change upon the first ligand binding to the active trimer of dimers. *L* is the ligand concentration. Therefore,

(6) $$\Delta E_{1}^{on,m}=-\text{log}\;\left(\frac{3k_{1}^{on,m}L}{k_{-1}^{on,m}}\right)=-\text{log}\;\left(\frac{3L}{K_{d1}^{on,m}}\right)$$

where $K_{d1}^{on,m}$ is the first ligand dissociation constant of an active trimer and $K_{d1}^{on,m}=k_{-1}^{on,m}/k_{1}^{on,m}$. Therefore, the free-energy level for the active trimer with one-ligand bound is

(7) $$E_{on}^{m}+\Delta E_{1}^{on,m}=E_{on}^{m}-\text{log}\;\left(\frac{3L}{K_{d1}^{on,m}}\right)$$

Similarly, we obtain the free-energy levels for the pure-type trimer of dimers, shown in Table 3.

Using Boltzmann’s law, the probability that the pure-type trimer of dimers is active is as below.

(8) $$p_{on}=\frac{1}{1+e^{\Delta f^{m}}}$$

(9) $$\Delta f^{m}=E_{on}^{m}-E_{off}+\text{log}\;\left(\frac{1+\frac{3L}{K_{d1}^{off,m}}+\frac{3L^{2}}{K_{d1}^{off,m}K_{d2}^{off,m}}+\frac{L^{3}}{K_{d1}^{off,m}K_{d2}^{off,m}K_{d3}^{off,m}}}{1+\frac{3L}{K_{d1}^{on,m}}+\frac{3L^{2}}{K_{d1}^{on,m}K_{d2}^{on,m}}+\frac{L^{3}}{K_{d1}^{on,m}K_{d2}^{on,m}K_{d3}^{on,m}}}\right)$$

Now, we consider a receptor cluster with *n* trimers of dimers. We assume that the energy for a cluster depends linearly on the number of trimers, that is, the free-energy level for the cluster to be in a certain state is the sum of the free-energy level for each trimer of dimers to be in the same state. Thus, the probability of the *n*-trimer cluster being active at equilibrium is

(10) $$p_{on}=\frac{1}{1+e^{n\Delta f^{m}}}$$

Finally, we consider a cluster of mixed-type receptors. Here, we work on the case of two types, Tar and Tsr, as an example. In this case, a trimer could contain three Tar homodimers, three Tsr homodimers, or a combination of Tar and Tsr homodimers. For simplicity, we use the approximation wherein only trimers made of the same homodimers exist in a mixed-type receptor cluster. With this assumption, a Tar-Tsr cluster only consists of the pure Tar trimers and the pure Tsr trimers. We only consider the response of the mixed-type cluster to a single type of ligands. The probability of the mixed-type receptor cluster with *n_a_* Tar trimers and *n_s_* Tsr trimers coupled being active at equilibrium is

(11) $$p_{on}=\frac{1}{1+e^{(n_{a}\Delta f_{a}^{m}+n_{s}\Delta f_{s}^{m}}}$$

(12) $$\Delta f_{a}^{m}=E_{on,a}^{m}-E_{off,a}+\text{log}\;\left(\frac{1+\frac{3L}{K_{d1,a}^{off,m}}+\frac{3L^{2}}{K_{d1,a}^{off,m}K_{d2,a}^{off,m}}+\frac{L^{3}}{K_{d1,a}^{off,m}K_{d2,a}^{off,m}K_{d3,a}^{off,m}}}{1+\frac{3L}{K_{d1,a}^{on,m}}+\frac{3L^{2}}{K_{d1,a}^{on,m}K_{d2,a}^{on,m}}+\frac{3L^{2}}{K_{d1,a}^{on,m}K_{d2,a}^{on,m}K_{d3,a}^{on,m}}}\right)$$

(13) $$\Delta f_{s}^{m}=E_{on,s}^{m}-E_{off,s}+\text{log}\;\left(\frac{1+\frac{3L}{K_{d1,s}^{off,m}}+\frac{3L^{2}}{K_{d1,s}^{off,m}K_{d2,s}^{off,m}}+\frac{L^{3}}{K_{d1,s}^{off,m}K_{d2,s}^{off,m}K_{d3,s}^{off,m}}}{1+\frac{3L}{K_{d1,s}^{on,m}}+\frac{3L^{2}}{K_{d1,s}^{on,m}K_{d2,s}^{on,m}}+\frac{L^{3}}{K_{d1,s}^{on,m}K_{d2,s}^{on,m}K_{d3,s}^{on,m}}}\right)$$

**Figure 7:**
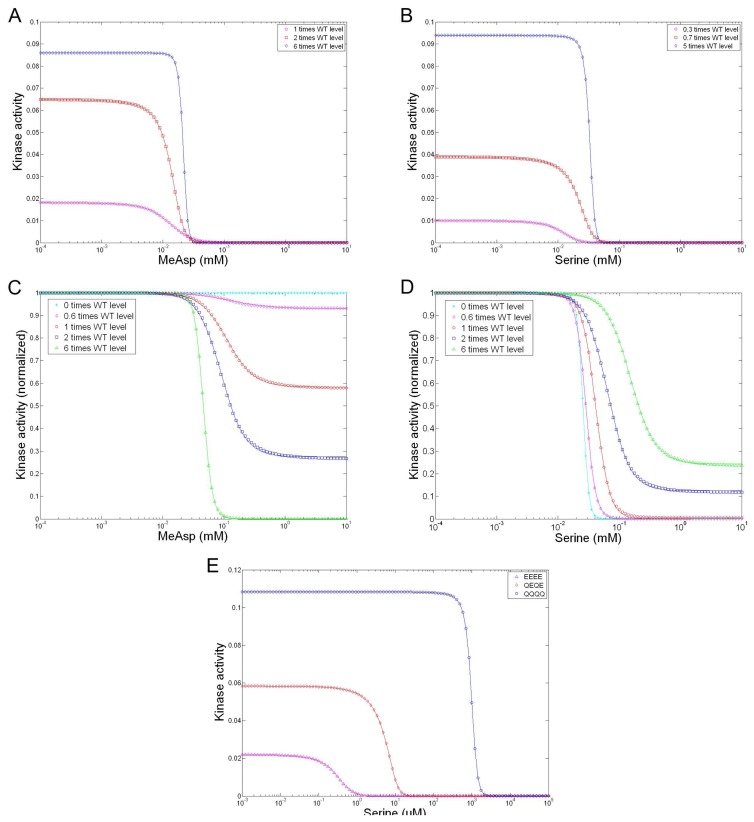
Responses of receptor Tsr *in vitro* and *cheRcheB* mutants with varied expression levels of Tar or Tsr. (**A**) Simulated responses to MeAsp of *cheRcheB* mutant cells expressing only Tar at 1 (○), 2 (□) and 6 (◇) times the native level; (**B**) Simulated responses to serine of *cheRcheB* mutant cells expressing only Tsr at 0.3 (○), 0.7 (□) and 5 (◇) times the native level; (**C**) Simulated responses to MeAsp of *cheRcheB* mutant cells expressing Tsr at the native level and Tar at 0 (*), 0.6 (◇), 1 (○), 2 (□) and 6 (△) times the native level; (**D**) Simulated responses to serine of *cheRcheB* mutant cells expressing Tsr at the native level and Tar at 0 (*), 0.6 (◇), 1 (○), 2 (□) and 6 (△) times the native level; (**E**) Simulated responses to serine by the receptor Tsr at the methylation state QQQQ (○), QEQE (◇) and EEEE (△).

We use this model to explain the observed ultrahigh cooperativity. We simulate the responses to methyl-aspartate (MeAsp) and serine in the *cheRcheB* mutants with different expression levels of the receptors Tar and Tsr and compare to Sourjik and Berg’s experiments. In Figure 7(A) are shown the responses to MeAsp in the *cheRcheB* mutants with only Tar expressed (compare to Figure 2(a) in [39]), in Figure 7(B) are the responses to serine with only Tsr expressed (compare to Figure 2(b) in [39]), in Figure 7(C) are the responses to MeAsp with native-level Tsr and varied-level Tar expressed (compare to Figure 1(c) in [39]), and in Figure 7(D) are shown the responses to serine with native-level Tar and varied-level Tsr expressed (compare to Figure 1(d) in [39]). We also simulate the kinase activity responses of Tsr to serine *in vitro*, shown in Figure 7(E) and compare to Li and Weis’ experiments (Figure 3 in [67]). Data fitting with the Hill function shows that the simulation results have quantitative agreement with the measures. The details of the simulations, such as parameter values and data fitting, are omitted here but documented in [145]. Our modeling on a single trimer of dimers and extension to a cluster of trimer of dimers indicates that the strongly-coupled trimer of dimers is the core unit for signaling function, that the short-range interaction between dimer members of a trimer, which we call the *intratrimer interaction*, plays a key role, and that the long-range interaction between trimers in a loosely coupled cluster, which we call the *intertrimer interaction*, is responsible for ultrahigh cooperativity.

## 5. Spontaneous Spatial Pattern Formation in Populations

At the population level, cell-cell-signaling and chemotaxis provide a mechanism for long-range cell-cell communication and formation of multicellular spatial patterns [9,146–152]. The spectacular growth patterns observed in cultures provide a simple and manipulable system for studying bacterial population chemotaxis in more complex environments, such as in biofilm formation, and in bioremediation, which is a process that uses microorganisms to remove pollutants [153,154]. The excitation and adaptation response of bacteria are crucial in the formation of these patterns. In this section, we review biological experiments and mathematical models for the patterns formed by cells such as *E. coli* that use a run-and-tumble strategy. Pattern formation in other bacterial colonies, e.g., *Bacillus*, *Proteus* and *Myxococcus*, and mathematical models of more complicated biofilms are discussed in [155–165].

### 5.1. Pattern Formation in Bacterial Colonies

Chemotaxis of *E. coli* can lead to spontaneous, self-organized pattern formation. In the 1960’s it was found that a *E. coli* colony forms moving bands/rings when exposed to a nutrient that also serves as a chemoattractant [167]. In the 1990’s, it was shown that *E. coli* colonies can organize into stable spatial patterns in either a semi-solid agar or liquid medium, by responding to a self-secreted chemoattractant (aspartate and analogues) [9,146]. When grown in semi-solid agar with a single nutrient source (e.g., succinate), a droplet of *E. coli* grows and depletes nutrient locally, and over the following three days, a concentric swarm ring forms and spreads radially from the inoculation site. A symmetric array of spots or stripes may form sequentially in the wake of the spreading ring depending on the initial nutrient concentration [9,146]. The speed of the swarm ring is observed to be inversely proportional to the initial nutrient concentration. When grown in a liquid medium, *E. coli* cells secrete attractant and self-organize into network patterns initially, and the networks subsequently collapse into moving aggregates which merge over time. The timescale of patterning for the liquid suspension experiments is of the order of minutes. Mittal *et al*. [166] studied *E. coli* aggregate formation in a quasi-2D system, and showed that the size of an aggregate is about 150 ~ 200 *μ*m in diameter and depends primarily on the adaptation time scale of the bacteria and only weakly on the total number of cells. Recently, *E. coli* traveling pulse patterns have also been observed in more complicated nutrients [168,169]. Due to the complicated nature of the nutrient, the exact chemical signal that leads to these patterns is not known, although the mechanism is believed to be similar to Budrene-Berg experiments. The adoption of the recent microfluidic techniques, allows precise tracking of individual trajectories of cells and analyze their movement in great detail. In [169], it is found that not only the mean run length of cells but also the directional persistence are larger in the direction of wave propagation. By incorporating these asymmetries of cell movement to a kinetic model similar to the hybrid cell-based model discussed later, these authors matched numerical simulations to experimental data seamlessly.

The enteric bacterium *P. mirabilis*, which is a pathogen that forms biofilms *in vivo* and has a similar chemotactic system to *E. coli*, can swarm over hard surfaces and form a variety of spatial patterns in colonies. It has been shown that *P. mirabilis* colonies, when grown on hard surfaces, can grow and expand, and form radial and spiral stream patterns in the center of the colony [170]. Remarkably, the spiral streams always wind counterclockwise in repeated experiments. Xue *et al.* [170] showed that assuming that cell secrete a chemoattractant, the formation of streams can be explained as a result of the instability induced by local production of attractant by cells, and the spiral rotation of the population results from the fact that cells swim with a rightward bias when moving close to a surface [171,172].

### 5.2. Mathematical Models of E. coli Pattern Formation

The formation of these bacterial patterns involves a complex interplay between different processes, including consumption of nutrients, production of chemoattractants, tactic movement towards the attractant, and hydrodynamic interaction with the environment. Moreover, formation of these spatial patterns can involve millions of cells. Mathematical models of the patterns include continuum models that incorporate these processess in a phenomenological way, and hybrid cell-based models that allow detailed description of the microscopic behavior. The continuum models are easier to implement numerically and more amenable to mathematical analysis, but justification of these models has to be addressed. Hybrid cell-based models can be used to integrate better descriptions of the experimental picture, but can be computationally expensive.

Continuum approaches to bacterial pattern formation

A variety of Patlak-Keller-Segel type systems

(14) $$\begin{array}{l} \frac{\partial n}{\partial t}=\nabla \cdot (D_{n}\nabla n-\chi (S)n\nabla S)+f(n,S,F)\\ \frac{\partial S}{\partial t}=D_{s}\Delta S+g_{s}(n,S,F)\\ \frac{\partial F}{\partial t}=D_{f}\Delta F+g_{f}(n,S,F) \end{array}$$

have been developed and applied to model *E. coli* patterns [147,173–182]. Here *n* is the cell density, *S* is the concentration of the extracellular chemical, *F* is nutrient, *D_n_*, *D_s_* and *D_f_* are the diffusion coefficients, *χ*(*S*) is the chemotaxis sensitivity, and *f*(*n, S, F*), *g_s_*(*n, S, F*) and *g_f_* (*n, S, F*) represent the local dynamics. The first equation in the system is called the Patlak-Keller-Segel chemotaxis equation, or PKS equation for short. Mathematical properties of the system (14) have been studied, for example in [182], and also see [183,184] for recent reviews.

Traveling wave solutions of the system (14) have been studied extensively as a means of describing the moving band formation observed in *E. coli* colonies. Keller and Segel [173] first applied the chemotaxis model without the *F* equation and cell growth term to describe Adler’s experiments of *E. coli* chemotactic band formation. They showed that the system of equations allow for an analytic traveling wave solution, but they required a singular chemotaxis sensitivity which violates the fact that cells have finite speed when *S* becomes small. A nonsingular chemotactic sensitivity, however, would lead to a moving band with decreasing speed and broadening density profile due to the diffusive effect of cell run-and-tumble. Later [174,175] showed numerically that by incorporating cell growth and death the shape and the speed of the band can be stabilized. More recent work based on the velocity-jump process described later has substantially clarified the picture [168].

Numerous extensions of the PKS model at system 14 have been proposed to model the *E. coli* swarm ring and aggregation patterns found by Budrene and Berg [9,146]. Ben-Jacob *et al.* [176] and Tsimring *et al*. [177] added a repellent field that was assumed to autocatalyze the production of attractant but to date there is no experimental confirmation of a repellent. In a model proposed by Polezhaev *et al.* [181] cells become immobile upon starvation, and simulations of the model predict the formation of a swarm ring and stable aggregation patterns composed of immobile cells. However, the swarm ring formed has a very diffuse front, in contrast to what is observed experimentally. The above models can produce patterns with some similarity to those in the semi-solid agar experiments, but how different processes control the patterns is not clear. To address this, Brenner *et al.* [185] analyzed a model of the form system (14), and suggested that movement of the swarm ring was driven by local nutrient depletion., The integrity of the swarm ring results from the high attractant concentration at the ring, whereas aggregates are suggested to form within the ring through fluctuations about the unstable uniform cell density. In a subsequent paper [186] an analysis of the transformation of high cell density cylinders into regularly spaced aggregates from the swarm ring was undertaken, with the conclusion that a shifting balance between diffusion and chemotaxis leads to collapse of the strands. Despite numerous attempts, there is at present no complete understanding of the pattern-forming process in *E. coli*.

#### 5.2.1. Hybrid Cell-based Models

Hybrid cell-based models for bacterial pattern formation have also been developed [170,187,188]. In these models, each cell is characterized by its position **x** ε ℝ*^N^*, velocity **v** ε *V* ⊆ ℝ*^N^*, internal state y and other auxillary variables indicating the metabolic state of the cell. The movement of each cell is modeled as a velocity jump processes with instantaneous velocity jumps mimicking the relatively short tumbles. Because in the aformentioned experiments, the average volume fraction of the cell population in the substrate is small, these models assumed that cells are well separated with no mechanical interactions between them, which means that cell movements are independent of each other. The rate of velocity jumps depends on the internal variables which describe intracellular signaling, and this can lead to large systems for the internal network. To reduce computational time, and with the goal of understanding the role of excitation and adaptation of cell signaling to the population behavior, the abstract linear cartoon model discussed earlier was used to describe the excitation and adaptation components of cell signaling,

(15) $$\begin{array}{l} \frac{{dy}_{1}^{i}}{dt}=\frac{G(S({\text{x}}^{i},t))-(y_{1}^{i}+y_{2}^{i})}{t_{e}}\\ \frac{{dy}_{2}^{i}}{dt}=\frac{G(S(x^{i},t))-y_{2}^{i}}{t_{a}} \end{array}$$

Here the superscript *i* is the index of the cell, *S* is the local attractant concentration and *t_e_* and *t_a_* (with *t_e_* ≪ *t_a_*) are constants defining excitation and adaptation timescales. The function *G*(*S*) models the detection of the extracellular (chemoattractant) signals. To model the run and tumble movement, these models describe the velocity jumps using a turning kernel *T* and a turning rate *λ* for each cell, given by forms similar to the following

(16) $$T=\frac{1}{\left|V\right|}\;\;\;\text{and }\;\;\lambda^{i}=\lambda_{0}(1-\frac{y_{1}^{i}}{\gamma_{0}+|y_{1}^{i}|})$$

Since particles are conserved in the turning process

$$\int_{v}T(v,v^{\prime })\;dv=1$$

The foregoing individual-based model for cell movement can be coupled with continuum reaction-diffusion equations to describe the evolution of the extracellular nutrient and attractant concentrations. The combined system is solved with a hybrid scheme in which the movement of each cell is simulated by a Monte Carlo method while the reaction-diffusion equations are solved with an alternating direction method. The details of this algorithm can be found in [189].

**Figure 8:**
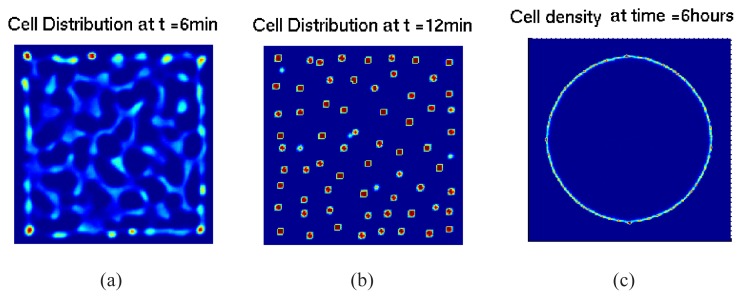
Simulated *E. coli* patterns by a cell-based model. (**a**) Network formation from an uniform cell lawn; (**b**) Aggregate formation from the network; (**c**) Traveling wave formation from a single inoculum in the center. Adapted from [187] with permission.

To model the experiments done by Budrene and Berg, the hybrid cell-based model was coupled with the following equations for the attractant and nutrient concentrations in [187]

(17) $$\begin{array}{l} \frac{\partial S}{\partial t}=D_{s}\Delta S+\gamma \sum_{n=1}^{N}\delta (x-x^{i})+\mu \\ \frac{\partial F}{\partial t}=D_{f}\Delta F-k\sum_{n=1}^{N}\delta (x-x^{i})\\ S(x,0)=0\\ F(x,0)=f_{0} \end{array}$$

where *D_s_* and *D_f_* are the diffusion rates, *γ* defines the secretion rate of attractant by cells, *k* is the consumption rate of the nutrient, and *μ* is an unspecified degradation rate. Simulations of the hybrid cell-based model can predict the time sequence of the network and aggregate formation in liquid medium and swarm ring formation in agar as in the experiments of Budrene and Berg (*cf.* Figure (8)). In particular, these simulations can reproduce the sharp wave front of the swarm rings, in agreement with experiments.

To model the traveling band formation observed by Adler, the hybrid model was coupled with the following equation for *S* [188],

(18) $$\begin{array}{l} \frac{\partial S}{\partial t}=D_{s}\Delta S-\gamma \sum_{n=1}^{N}\delta (x-x^{i})\\ S(x,0)=1 \end{array}$$

where *γ* is the consumption rate of the signal S by cells. Here *γ* becomes negative because in this set of experiments, the signal becomes the nutrient source of the cells and they consume it instead of secrete it. It was found that the cell population moves towards higher concentrations of signal at a constant speed, and the profile of the traveling cell population shows a dropout phenomena which has not been reported by any continuum model [188]. When coupled with cell growth, the wave shape and speed can be stabilized, but oscillations of the wave speed have been observed in the hybrid model [190].

**Figure 9:**
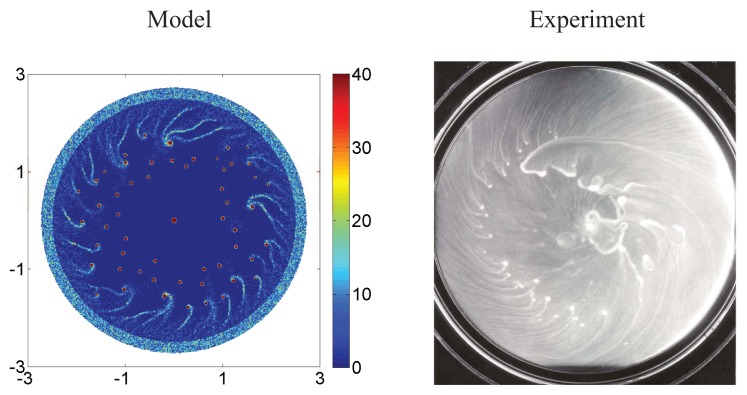
Spiral streams in a growing *Proteus mirabilis* colony. Reproduced from [170] with permission.

The hybrid cell-based model has also been applied to model radial and spiral stream formation of *P. mirabilis*. The difference from the application to *E. coli* patterns is that in the patterns formed by *P. mirabilis*, cells move close to a surface, and thus have a clockwise bias in their movement when observed from above the cell looking toward the surface. To incorporate the bias, Xue *et al*. [170] added an angular component to the velocity of each cell. Remarkably, the hybrid cell-based model predicts the spiral stream patterns with the correct chirality qualitatively (Figure 9). Further parameterization of the model with refined experimental measurements are needed to obtain quantitative comparisons between experiments and modeling.

Hybrid cell-based models based on simplified descriptions of cell signaling have also been used to study *E. coli* population chemotaxis in well-controlled spatially and temporally varying signal fields [191,192]. Quantitative agreement of the cell density profile between experiments and models were achieved by refining the parameters of these models with single cell data.

#### 5.2.2. From Cell-based Models to Continuum Models

The hybrid cell-based models described above can be used to integrate details on cell signaling and movement faithfully. However, when used to simulate population behavior, it becomes computationally intensive, especially when some parameters of the model are unknown and parameter exploration is needed. The continuum models such as those based on PKS equations are computationally managable and analytically amenable. However, justification of these models under different scenarios of signals, and the relationship between macroscopic parameters in these models with parameters known in the signal transduction steps, are not rigorously established in the original PKS equation.

To fill in this gap, significant effort has been put in deriving continuum models from cell-based models for chemotaxis of *E. coli*. Early works derived the PKS equation from cell movement modeled as biased random walks with signal-dependent parameters [193–203]. Recently, the simplified description of Equation (15) of signal transduction has also been incorporated, and the PKS equation was derived when the signal detected by cells changes slow enough so that cells are close to their fully adapted state [189,204,205]. The derivation of the equation starts from linearization of the cartoon model of signal transduction around its adapted state, by introducing the equivalent variable *z̄* = **y** − (0*,G*(*S*))*^T^*, which satisfies

(19) $$\begin{array}{l} \frac{dz_{1}}{dt}=-\frac{z_{1}}{t_{e}}\\ \frac{dz_{2}}{dt}=\frac{-z_{1}-z_{2}}{t_{a}} \end{array}$$

and involves asymptotic approximations of the resulting master equation of the velocity jump process,

(20) $$\begin{array}{c} \frac{\partial p(x,v,\bar{z},t)}{\partial t}+\nabla_{x}\cdot \left(vp(x,v,\bar{z},t)\right)+\nabla_{z_{1}}\cdot \left[\left(-\frac{z_{1}}{t_{e}}\right)p(x,v,\bar{z},t)\right]+\nabla_{z_{2}}\cdot \left[\left(\frac{-z_{1}-z_{2}}{t_{a}}\right)p(x,v,\bar{z},t)\right]\\ =\lambda (z_{1})\left(-p(x,v,\bar{z},t)+\int_{V}T(v,v^{\prime })p(x,v^{\prime },\bar{z},t)dv^{\prime }\right) \end{array}$$

Here *p*(**x***,***v***, z̄, t*) is the probability density for a cell to be at position **x** ε ℝ*^N^*, with velocity **v** ε *V* ⊂ ℝ*^N^*, and intracellular state *z̄* ε ℝ*^q^*. The formal derivation of the approximation involves rescaling of Equation (20) on the diffusion space and time scales, calculating the internal state moment equations, and closing the infinite moment system. When the signal gradient is small, the adaptation time scale of the cell is much smaller than the time scale for signal variation, and the ratio of these times scales becomes a natural small parameter for closing the infinite moment system and applying perturbation methods to the closed moment system. This leads to a hierarchy of approximations of the lowest order moments such as the cell density *n*(x*, t*) = *∫ p*d**v**d*z̄*. The first order closure of *n*(**x***, t*) satisfies the following PKS equation [189],

(21) $$\frac{\partial n}{\partial t}=\nabla \cdot \left(\frac{s_{0}^{2}}{N\lambda_{0}}\nabla n-\frac{bs_{0}^{2}t_{a}}{N\lambda_{0}(1+\lambda_{0}t_{a})(1+\lambda_{0}t_{e})}\nabla S\right)$$

where *s*_0_ is the cell speed, *N* is the space dimension, λ_0_ is the basal turning rate, and *b* is the derivative ∂*_z_*__1__λ(0).

**Figure 10:**
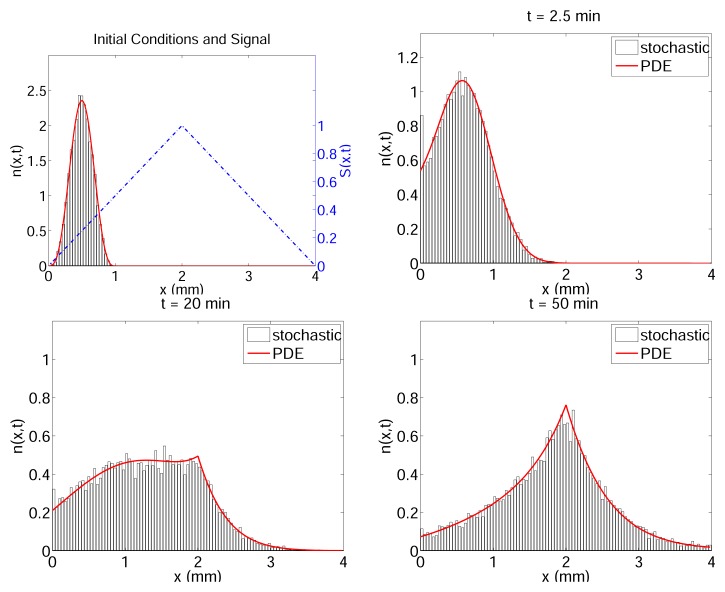
Comparison of solutions of the derived PDE (21) with stochastic simulations of the cell-based model. Parameters used: *s*_0_ = 20*μ/*s, *λ*_0_ = 1, *b* = 4. 10^4^ cells are used for the cell-based model. Bars: histogram of the cell positions computed from the cell-based model with a total number 10^4^ cells. Red lines: numerical solutions of Equation (21).

This PKS equation, in which the chemotactic sensitivity is defined in terms of cell parameters, provides a very good approximation to the spatial-temporal dynamics observed in the cell-based models under a variety of signal regimes, as long as the signal gradient times the cell is small compared to the reciprocal of the adaptation time [189]. Figure 10 shows a typical comparison between the results of a stochastic simulation of the cell-based model with numerical solutions of Equation (21) for a given piecewise linear signal with a peak at *x* = 2*mm*.

However when the signal changes rapidly along the cell’s trajectory, the underlying assumption of the derivation is not satisfied and Equation (21) is not adequate to describe the population dynamics [170]. The main reason is that due to fast signal variation, intracellular signaling is far from its adapted state, and this derivation has to be accounted for in a continuum model by introducing new variables such as higher-order moments of the internal states. Recently, such a continuum model has been proposed by including a mean methylation level of receptors [206], and the model can be used to capture certain macroscopic quantities of the cell population such as the mean position of a colony compared to experiments and cell-based simulations. However, matching between solutions of the proposed continuum model and underlying cell-based model/experimental data as spatial-temporal functions was not achieved in a satisfactory manner. Thus deriving continuum models for bacterial chemotaxis that can quantitatively predict the evolution of cell density in large signal gradients is still an open problem.

## 6. Discussion

The signal transduction pathway that governs bacterial chemotaxis in *E. coli* is one of the most thoroughly understood molecular systems. At the single-cell level it involves excitation and adaptation responses to signals, which enable cells to move to more favorable environments. At the population level, it provides a mechanism for cell-cell communication by secretion of the attractant and for formation of multicellular structures. To understand these phenomena at different spatial and temporal scales quantitatively, biologists, physicists, and mathematicians have been involved in experimentation and modeling. Although much is known, there are still many questions to be addressed.

At the receptor homodimer level, there are questions concerning conformational changes of the cytoplasmic domain for signaling, especially in the HAMP region and the signaling region. At the trimer of dimers level, further studies to determine the stoichiometry of the ternary MCP-CheA-CheWsignaling complexes, and how the signaling-induced conformational changes of one homodimer affect other dimer members within a trimer, are needed. Major questions remain at the receptor cluster level, where a cluster of trimers of dimers is probably used. One hypothesis that could be tested in this context is that for short-range interaction among three dimers of the same trimer, the protein interaction is primarily due to the direct coupling of dimers in the cytoplasmic domain, while for longer-range interaction among trimers (dimers of different trimers), the protein interaction is due to indirect coupling through the interconnected CheA and CheW network, or possibly membrane-mediated elastic interaction.

At the population level open questions include: how to derive a quantitative macroscopic description of bacterial population chemotaxis when the external signal changes rapidly, or whether such an approach is even possible. Currently continuum models are only derived from coarse-grained or abstract models of signal transduction that can perform excitation and adaptation. Derivation of continuum models from cell-based models that take into account detailed descriptions of cell signaling is currently under investigation and will be published elsewhere.

## Figures and Tables

**Table 1:** Structure-function relationship of chemoreceptor clusters in *E. coli* chemotaxis.

	Dimer	Trimer of dimers	Cluster of trimers
**Ligand binding**	Yes minimal structural unit	Yes	Yes
**Adaptational modification**	Yes minimal structural unit	Yes	Yes
**Transmembrane signaling**	Yes minimal structural unit	Yes	Yes
**Kinase activity control**	No	Yes minimal structural unit, core functional unit (maximal kinase activation)	Yes
**Cooperativity**	Low Hill coefficient~1	Moderate Hill coefficient~2-3; in wild-type cells and some *cheR/cheB/cheRcheB* mutant strains	High Hill coefficient≫3; in *cheRcheB* mutant strains with Tar or Tsr highly overexpressed, in receptor Tsr *in vitro*

**Table 2:** Mathematical models of bacterial chemotaxis (1982–2012).

Excitation, adaptation, and robustness		
Model	Methods	Assumptions and Outcomes
Goldbeter and Koshland Jr [91]	ODE	Includes ligand binding and one-site methylation; Uses two-state assumption (methylated and demethylated); Demonstrates that perfect adaptation could be achieved via methylation whose reaction rates depend on receptor occupancy.
Block *et al.* [10]	ODE	Uses two-state assumption (CW and CCW); Includes adaptation; Demonstrates that transition between the run and tumble states depends on adaptation to the sensory input.
Asakura and Honda [78]	ODE	Includes ligand binding and multiple-site methylation; Uses two-state assumption (methylated and demethylated); Shows adaptation to attractants and repellents at both low and high background concentrations via multiple methylation.
Segel *et al.* [92]	ODE	Similar with Goldbeter and Koshland Jr [91] but allows receptor modification to occur on both ligand-free and ligand-bound receptors.
Bray *et al.* [93]	ODE	Includes ligand binding, phosphorylation cascade, and motor control; Reproduces the motor bias response to step changes in attractants and repellents ; Does not include methylation/demethylation and model for adaptation.
Bray and Bourret [94]	ODE	Models the ternary MCP/CheA/CheWsignaling complex formation and adds it into Bray *et al.* [93] to study the effect of the signaling complex formation on motor bias.
Hauri and Ross [113]	ODE	Models the complete signal transduction pathway and reproduces the excitation and adaptation phases of bacterial chemotaxis in the experimentally agreed timescales; Assumes that CheA autophosphorylation rate dependent on the methylation level of receptors.
Spiro *et al.* [75]	ODE	Models the complete signal transduction pathway with reduced three methylation states and reproduces excitation and adaptation in the experimentally agreed timescales. Assumes the autophosphorylation rate increases with the methylation level, the methylation rate is greater for attractant-bound than attractant-free receptors, and the demethylation rate is independent of ligand binding of receptors.
Barkai and Leibler [96]	ODE	Includes ligand binding and methylation/demethylation for a three-component system (MCP, CheR and CheB); Uses two-state assumption (active or inactive for receptors); Assumes that CheR works at saturation in a constant rate and CheB acts only on active receptors in a rate independent of ligand binding; Shows perfect adaptation of receptor activity and robustness of the ratio of adapted steady-state receptor activity over prestimulus activity for a wide range of parameter values.
Levin *et al.* [114]	ODE	Investigates the effect of changes in chemotactic protein expression levels on the concentration of CheYp, and compares the fine-tuned and the robust adaptation models in this aspect.
Morton-Firth and Bray [95]	Free-energy-based stochastic simulation	Includes phosphorylation cascade; Simulates the temporal fluctuation of CheYp.
Morton-Firth *et al.* [99]	Free-energy-based stochastic simulation	Includes phosphorylation cascade (based on [95]) and methylation/demethylation (based on [96]); Assumes that CheR only methylates inactive receptors and CheBp only demethylates active receptors; Shows excitation and adaptation;
Yi *et al.* [81]	ODE	Analyzes the Barkai and Leibler’s model and shows an integral feedback control imbedded in the system that leads to robust perfect adaptation.
Almogy *et al.* [115]	ODE	Proposes an alternative adaptation mechanism that is through dephosphorylation of CheYp by both CheZ and the CheAs–CheZ complex rather than methylation/demethylation of receptors.
Mello and Tu [116]	ODE	Studies the robust adaptation problem analytically and proposes six conditions for achieving perfect adaptation, confirming those key assumptions that Barkai and Leibler use [96].
Arocena and Acerenza [117]	ODE	Studies the response range of bacterial chemotaxis, and shows the wider range when receptor modification is through methylation and phosphorylation than through attractant binding.
Kollmann *et al.* [111]	ODE	Uses a simplified signaling network only including a single methylation site; Shows the robustness to the intercellular variation in chemotactic protein concentrations arising from gene expression, and the variation of CheYp is much smaller than that of other proteins.
Tu *et al.* [112]	ODE, mean-field theory	Simulates chemotactic responses to time-varying exponential ramp, sine wave, and impulsive signals.
**Receptor clustering and signaling sensitivity**		
Bray *et al.* [118]	probability analysis	Conceptual model; Shows that receptor clustering and conformational spread among neighboring receptors can explain high sensitivity.
Shi and Duke [97]	statistical mechanics, Ising model	Ising-type model and mean-field theory applied; Shows that receptor coupling strength affects response more than attractant binding.
Duke and Bray [119]	Monte Carlo methods	Monte Carlo simulation of [97]; Shows higher signaling sensitivity than the uncoupled system and ability to respond to over five order of magnitude of attractant concentrations.
Shi [98]	statistical mechanics, Ising model	Adaptive Ising-type model with CheR, CheBp, and their negative feedback effect on receptor activity included; More robust than [97] because of relaxation of the filed strength assumptions; Shows high sensitivity.
Shi [120]	Ising model	Compares simulations of the models [97,98] with experiments and shows good agreement on the ratio of attractant binding to receptor-receptor interactions, the adaptation time, as well as the ratio of pre- and post-stimulus CheA phosphorylation.
Shi [121]	Ising model, Monte Carlo methods	Considers the receptor movement and allows them to float; Shows strong correlation for neighboring receptors and exponential decay with increasing receptor-receptor distance.
Levin *et al.* [122]	Monte Carlo methods	Studies effect of binding and diffusion of CheR through receptor clusters with the model [99]; Shows that if binding is within the physiological limits, CheR can access and modify a large number of receptors in cluster.
Shimizu *et al.* [123]	Ising model, free-energy-based stochastic simulation	Ising model incorporated into [99]; Compares effect of receptor array size and geometry on sensitivity, gain and signal-to-noise ratio; Reproduces overshoot.
Mello and Tu [100]	Ising model	Deterministic version of Ising-type model; Includes receptor interactions between Tar and Tsr; Includes methylation/demethylation (same assumptions as [96,99]); Reproduces the FRET data on *cheR/cheB/cheRcheB* mutant and wild-type cells [15] using two different parameter sets.
Mello *et al.* [124]	Ising model, mean-field theory, Monte Carlo methods	Mean-field theory applied to and Monte Carlo simulation of [100].
Goldman *et al.* [101]	Lattice gas model, Monte Carlo methods	Applies 2-D lattice gas model of protein association to chemoreceptor clusters.
Sourjik and Berg [39]	MWC model	Applies MWC model to explain their FERT data.
Albert *et al.* [102]	ODE	Model for dynamic formation of trimer of dimers; Assumes the time scale of association and dissociation of trimer of dimers comparable to that of ligand binding and kinase activity, which was disproved later by experiments [103].
Rao *et al.* [130]	MWC model	Model of static trimer of dimers; Reproduces *in vitro* kinase activity data on Tar [104] and Tsr [67] as well as *in vivo* data on mutant cells [15]
Mello and Tu [107]	MWC model	Generalizes MWC model for allosteric interaction and multiple signal integration in heterogeneous receptor clusters; Reproduces measured responses for 14 mutant strains with varied expression levels of Tar and/or Tsr [39].
Keymer *et al.* [106]	MWC model	Proposes two regimes for a two-state receptor: regime I is characterized by low to moderate kinase activity and a low, constant inhibition number for half-maximal activity *K_i_*, in which coupling of receptors leads to high sensitivity (in the case of wild-type and *cheR* mutant cells); regime II is characterized by high kinase activity and a high *K_i_*, increasing with the methylated level of receptors, in which coupling leads to high cooperativity (in the case of *cheRcheB* mutant cells); Accordingly proposes a modified MWC model; Reproduces Sourjik and Berg’s FRET data [15].
Endres and Wingreen [110]	MWC model	Adaptation model based on ‘assistant-neighborhoods’ [105], using the key assumptions on CheR and CheBp as [96,99]; Incorporates the MWC model [106]; Shows sensitivity and adaptation for mixed-type receptors observed in [15]; Suggests two limits of adaptation to attractants: (1) saturation of ligand binding sites on receptors; (2) full methylation of receptors.
[129]	MWC model, Ising model	Compares activity response of receptor clusters generated by one-dimensional Ising-type model, two-dimensional Ising-type model, and two-regime MWC-type model; Shows that the outputs of Ising-type models are not consistent with the FRET data on activity responses to steps of attractants for wild-type and *cheR* mutant cells [15], which the MWC-type model can reproduce; Suggests strongly-coupled receptor clusters.
Mello and Tu [126]	MWC model	Studies the mechanism how the cells maintain high sensitivity over a wide range of backgrounds based on a simplified version of [107] for homogeneous receptor complexes.
Endres *et al.* [131]	statistical mechanics, MWC model	Model of static trimer of dimers; Reproduces *in vitro* kinase activity data on Tar [104,108,109].
Park *et al.* [132]	sensitivity analysis	Performs sensitivity analysis for trimer of dimers and shows enhanced signaling sensitivity compared with dimers.
Hansen *et al.* [127]	MWC model	Robust adaptation model extended from [110] including binding and unbinding of CheR and CheBp; Analyzes adaptation limits from the angle of CheR and CheB kinetics.
Endres *et al.* [133]	MWC model, statistical method	Determines the sizes of signaling clusters through best fitting *in vivo* FRET data with the model [106] using statistical PCA method; Shows the cluster sizes increasing with methylation levels.
Hansen *et al.* [134]	statistical mechanics, MWC model	Model of dynamic signaling clusters of trimers of dimers, the boundaries of which are variable in simulation; Shows stronger coupling of active trimers of dimers than inactive.
Meir *et al.* [128]	MWC model, ODE	Analyzes the characteristics of precise adaptation and finds the asymmetries (*i.e*., different adaptation time) in responses to addition and removal of attractants; Proposes two possible sources of the asymmetry: (1) dynamic phosphorylation of CheB and (2) scarcity of methylation site.
Clausznitzer *et al.* [142]	MWC model, ODE	Studies the dynamics (time courses) of adaptation and evaluate the existing adaptation models.
Khursigara *et al.* [53]	MWC model	Study with experiments and simulations combined; A cutoff distance used to determine the range of interacting receptors and the size of signaling receptor clusters variable; Shows that the size of receptor arrays is relatively stable, non-correlated with the protein expression level, and the packing density is slightly varied in difficult growth media.
Xin and Othmer [143]	ODE	Model of trimer of dimers; Simulates dynamics for the overall pathway; Explains a line of *in vitro* kinase activity data on Tar and Tsr [67,104,108,109] and *in vivo* FRET data in mutant cells [15] with the single trimer without higher-order clusters; Shows enhanced sensitivity and robustness to protein expressions generated by trimer of dimers.
**Other features**		
Rao *et al.* [138]	ODE	Compares signaling pathways between *E. coli* and *Bacillus subtilis*; Shows robust adaptation in both pathways but *B. subtilis* can perform methylation-independent chemotaxis because of existence of CheV-CheY pathway.
Lipkow *et al.* [135]	spatiotemporal stochastic simulation	3D stochastic simulation of CheY phosphorylation, CheY/CheYp diffusion, CheYp binding to FliM and dephosphorylation; Studies effects of CheZ localization, motor position, and macromolecular crowding on spatial concentration of CheYp; Shows a constant concentration of CheYp throughout the cytoplasm when CheZ is restricted to anterior ends and an exponential gradient across the length of the cell when CheZ diffuses freely.
Lipkow [136]	spatiotemporal stochastic simulation	Studies the effect of CheZ localization; Suggests that clustering of CheZ–CheAs–CheYp at the cell poles, introducing a negative feedback to the CheYp level, serves a secondary adaptation mechanism and explains the overshoot of CheYp in *cheRcheB* mutant cells.
Endres [137]	statistical mechanics	Free energy-based model for formation of clusters of trimer of dimers; Studies the determining factors of the size of polar receptor clusters.
Roberts *et al.* [139]	ODE	Develops a control engineering method and applies it to elucidating the signaling pathways of *Rhodobacter sphaeroides* chemotaxis.
Tindall *et al.* [140]	ODE	Studies the signal integration mechanism in *Rhodobacter sphaeroides* chemotaxis.
Hamadeh *et al.* [141]	control theory	Studies the feedback configuration of *Rhodobacter sphaeroides*; Shows the role of cascade control in achieving robust functions.

**Table 3:** Free-energy levels for a pure-type trimer of dimers.

State	Free-energy Level (unit: *k_B_T*)
On with 0 ligand bound	$E_{on}^{m}$
On with 1 ligands bound	$E_{on}^{m}-\text{log}\;\left(\frac{3L}{K_{d1}^{on,m}}\right)$
On with 2 ligands bound	$E_{on}^{m}-\text{log}\;\left(\frac{3L^{2}}{K_{d1}^{on,m}K_{d2}^{on,m}}\right)$
On with 3 ligands bound	$E_{on}^{m}-\text{log}\;\left(\frac{L^{3}}{K_{d1}^{on,m}K_{d2}^{on,m}K_{d3}^{on,m}}\right)$

Off with 0 ligand bound	*E_off_*
Off with 1 ligands bound	$E_{off}-\text{log}\;\left(\frac{3L}{K_{d1}^{off,m}}\right)$
Off with 2 ligands bound	$E_{off}-\text{log}\;\left(\frac{3L^{2}}{K_{d1}^{off,m}K_{d2}^{off,m}}\right)$
Off with 3 ligands bound	$E_{off}-\text{log}\;\left(\frac{L^{3}}{K_{d1}^{off,m}K_{d2}^{off,m}K_{d3}^{off,m}}\right)$
